# Conditioned umbilical cord tissue provides a natural three-dimensional storage compartment as in vitro stem cell niche for human mesenchymal stroma/stem cells

**DOI:** 10.1186/s13287-016-0289-0

**Published:** 2016-02-11

**Authors:** Yuanyuan Yang, Catharina Melzer, Vesna Bucan, Juliane von der Ohe, Anna Otte, Ralf Hass

**Affiliations:** Department of Obstetrics and Gynecology, Biochemistry and Tumor Biology Laboratory, Hannover Medical School, Carl-Neuberg-Str. 1, Hannover, D-30625 Germany; Tongji Hospital Affiliated Tongji University, Shanghai, 200065 China; Department of Plastic, Hand and Reconstructive Surgery, Hannover Medical School, Carl-Neuberg-Straße 1, Hannover, D-30625 Germany; Department of Gynecology and Obstetrics, Biochemistry and Tumor Biology Laboratory, Hannover Medical School, Carl-Neuberg-Straße 1, Hannover, D – 30625 Germany

**Keywords:** Mesenchymal stroma/stem cells, Tissue conditioning, Umbilical cord tissue, Cryopreservation, Tissue engineering, Three-dimensional long-term culture

## Abstract

**Background:**

The use of large amounts of human multipotent mesenchymal stroma/stem cells (MSC) for cell therapies represents a desirable property in tissue engineering and banking in the field of regenerative medicine.

**Methods and results:**

Whereas cryo-storage of umbilical cord (UC) tissue pieces in liquid nitrogen without ingredients was associated with predominant appearance of apoptotic cells after thawing and re-culture, progressive growth of MSC was observed following use of cryo-medium. Moreover, conditioning of UC tissue pieces by initial explant culture and subsequent cryo-storage with cryo-medium accelerated a further MSC culture after thawing. These findings suggested that conditioning of UC tissue pieces provides an in vitro stem cell niche by maintenance of a 3-dimensional natural microenvironment for continuous MSC outgrowth and expansion. Indeed, culture of GFP-labeled UC tissue pieces was accompanied by increased outgrowth of GFP-labeled cells which was accelerated in conditioned UC tissue after cryo-storage. Moreover, cryopreserved conditioned UC tissue pieces in cryo-medium after thawing and explant culture could be cryopreserved again demonstrating renewed MSC outgrowth after repeated thawing with similar population doublings compared to the initial explant culture. Flow cytometry analysis of outgrowing cells revealed expression of the typical MSC markers CD73, CD90, and CD105. Furthermore, these cells demonstrated little if any senescence and cultures revealed stem cell-like characteristics by differentiation along the adipogenic, chondrogenic and osteogenic lineages.

**Conclusions:**

Expression of MSC markers was maintained for at least 10 freeze/thaw/explant culture cycles demonstrating that repeated cryopreservation of conditioned UC tissue pieces provided a reproducible and enriched stem cell source.

## Background

Mesenchymal stroma/stem cells (MSC) are recruited towards tissue injuries to exhibit repair, immune modulation, neovascularization, and differentiation capacity for support of tissue regeneration. Transplantation of MSCs for substitution of damaged cells or tissue as well as MSC expansion for tissue engineering in the field of regenerative medicine require appropriate cell sources which are provided by invasive collection of bone marrow-derived MSC [1] or adipose tissue-derived MSC [2] from appropriate biopsies. Alternatively, more easily accessible, noninvasive, and therefore ethically noncontroversial MSC sources are represented by neonatal tissues, including MSC from placenta or from umbilical cord (UC) [3, 4]. Previous studies revealed the advantages of explant cultures from human UC to obtain large quantities of human UC-MSC with long-term maintenance of stem cell properties [5].

Human MSC are characterized as a multipotent heterogeneous cell population which can be found in nearly all vascularized organs and tissues. The stem cell properties include self-renewal and regenerative potential [6]. Certain heterogeneity both in morphology and cell fate as demonstrated by isolation of MSC subpopulations [7] may result partially from interaction with neighboring cells in the cellular microenvironment, from altered trophic factors, pH, hypoxic conditions, or oxidative stress and provide difficulties in MSC characterization. Additional heterogeneous and even controversial findings provided a discussion platform for a clear description and definition of MSC [8]. Consequently, instead of a certain specific cell marker, a broad spectrum of simultaneous minimal criteria has been identified for MSC, including the capability for plastic adherence, paralleled by expression of the CD73, CD90, and CD105 surface molecules with concomitant absence of other cell type-specific markers such as CD14, CD31, CD34, CD45, and human leukocyte antigen DR (HLA-DR) [9, 10]. Furthermore, MSC exhibit migratory activities [11] and display at least a tri-lineage differentiation capacity along the osteogenic, chondrogenic, and adipogenic phenotype [9] whereby also trans-germline maturation pathways have been discussed. The differentiation potential of MSC together with immune-modulatory functions and an involvement in neovascularization represent multipotent cellular functionalities which are required for the use in regenerative medicine. MSC have thus been successfully applied in a variety of clinical trials – for example, large bone defects [12], cartilage lesions and traumatic articular cartilage defects [13], osteogenesis imperfecta [14], graft versus host disease [15], spinal cord injuries [16], cardiovascular diseases [17], and hematological pathologies [18] – indicating the need for large quantities of MSC.

A reproducible source for healthy stem cells including MSC could be represented by appropriately cryopreserved tissue. Previous work has demonstrated that cryopreservation of birth-associated tissues such as placenta and UC can serve for hematopoietic stem cells and MSC, respectively [19, 20]. Moreover, MSC can also be obtained after cryostorage of whole adipose tissue [21]. Several other studies using different fertility preservation strategies included embryo cryopreservation, oocyte vitrification, and ovarian tissue cryostorage [22]. In addition, counteracting certain mechanisms of post-thaw cell death could increase the quality of cryopreserved tissue [23]. In the present work, we demonstrate that conditioning of UC tissue pieces can minimize post-thaw cell death and serves as a reproducible MSC source and as a valuable three-dimensional storage compartment for repeatable cryopreservation and maintenance of MSC within their natural microenvironment.

## Materials and methods

### Cell and tissue culture of human UC and MSCs

The use of primary human MSC following explant culture from UC tissue was approved by the Ethics Committee of Hannover Medical School (Project #443) on 26 February 2009. Informed written consent was obtained from each patient.

MSC-like cells were isolated from human UCs as reported previously [5]. The cells were obtained from UC explant cultures of three different patients following delivery of full-term (38–40 weeks) infants either spontaneously or by Cesarean section. In brief, UC tissue was washed with phosphate buffer saline (PBS) to remove blood cells, cut into approximately 0.5 cm^3^ large pieces, and incubated in alpha-minimum essential medium (αMEM; Sigma Chemie GmbH, Steinheim, Germany) supplemented with 15 % allogeneic human AB-serum (HS; commercially obtained from blood bank, University Campus Lübeck, Germany), 100 U/ml penicillin, 100 μg/ml streptomycin, and 2 mM l-glutamine (Sigma) at 37 °C in a humidified atmosphere with 5 % carbon dioxide. After about 14 days of explant culture with fresh medium replacement every 3–4 days, the UC tissue pieces were removed and the adherent cells were harvested by accutase (Sigma) treatment for 3 minutes at 37 °C. The obtained cell suspension was centrifuged at 320 × *g* for 5 minutes and the cells were resuspended in MSC culture medium (αMEM supplemented with 10 % HS, 100 U/ml penicillin, 100 μg/ml streptomycin, and 2 mM l-glutamine) and subcultured in the appropriate passage.

The UC tissue pieces after initial explant culture were termed “conditioned” UC tissue. Conditioned tissue has been cultured for approximately 2 weeks allowing adaptation to the culture conditions in contrast to freshly prepared tissue. Cryopreservation of UC tissue was performed in cryomedium (90 % HS containing 10 % (v/v) dimethyl sulfoxide (DMSO)) with a freezing velocity of approximately 1 °C/minute (Nalgene Cryo 1 °C freezing container; Nunc: Wiesbaden, Germany) until the samples reached –80 °C. Thereafter, the cryopreserved UC tissues were stored in liquid nitrogen for 3 days until start of the next explant culture.

Green fluorescent protein (GFP) labeling of UC tissue pieces was performed by lentiviral transduction. Six UC tissue pieces of similar size were transduced with a third-generation lentiviral SIN vector containing the *eGFP* gene according to a labeling technique described previously for the transduction of MSCs [24]. Briefly, each of the six UC tissue pieces was separately centrifuged together with the lentivirus at 2000 × *g*/30 minutes, washed three times in MSC culture medium, and incubated in one well of a 24-well plate with 500 μl MSC medium. Fluorescence was measured at 480 nm excitation/520 nm emission wavelength using a Fluoroskan Ascent FL (Thermo Scientific, Waltham, MA, USA) after transfer of the labeled UC tissue pieces into one well of a 96-well microtiter plate, respectively. Following fluorescence measurement, the labeled UC tissue pieces were cultured further in the 24-well plates.

### Reverse transcription PCR analysis

Total RNA was extracted using the RNeasy Mini Kit (Qiagen, Hilden, Germany) according to the manufacturer’s instructions and reverse transcription RT-PCR was performed as described previously [25]. As a template, 2.5 μl cDNA was used with primers specific for CD73 (sense, 5′-CGC AAC AAT GGC ACA ATT AC-3′; antisense, 5′-CTC GAC ACT TGG TGC AAA GA-3′; amplification product 241 base pairs (bp)), CD90 (sense, 5′-GGA CTG AGA TCC CAG AAC CA-3′; antisense, 5′-ACG AAG GCT CTG GTC CAC TA-3′; amplification product 124 bp), CD105 (sense, 5′-TGT CTC ACT TCA TGC CTC CAG CT-3′; antisense, 5′-AGG CTG TCC ATG TTG AGG CAG T-3′; amplification product 378 bp), and eGFP (sense, 5′-CTA TAT CAT GGC CGA CAA GCA GA-3′; antisense, 5′-GGA CTG GGT GCT CAG GTA GTG G-3′; amplification product 165 bp). As a control, glyceraldehyde 3-phosphate dehydrogenase (GAPDH) PCR (sense, 5′-ACC ACA GTC CAT GCC ATC AC-3′; antisense, 5′-TCC ACC ACC CTG TTG CTG TA-3′; amplification product 452 bp) was performed (all primers customized by Eurofins, MWG GmbH, Ebersberg, Germany). Aliquots of 25 μl of each RT-PCR product were separated on a 2 % agarose gel including the standard GeneRuler 100 bp DNA Ladder (Thermo Scientific) and visualized by GelRed™ (Biotium Inc., Hayward, CA, USA) staining.

### Cell cycle analysis

The cell cycle analysis in the different MSC populations was performed as described previously [26]. Briefly, 5 × 10^5^ cells were fixed in 70 % (v/v) ice-cold ethanol at 4 °C for 24 hours. The fixed cells were stained with the CyStain DNA 2 step kit (Partec GmbH, Münster, Germany) and filtered through a 50 μm filter. Thereafter, samples were analyzed in a Galaxy flow cytometer (Partec) using the FloMax analysis software (Partec).

### Senescence-associated β-galactosidase assay

Cell senescence can be detected by different expression levels of senescence-associated β-galactosidase (SA-β-gal) [27]. The amount of senescent cells was determined using the SA-β-gal staining kit (Cell Signaling Technology, Danvers, Massachusetts, USA) and 4′,6-diamidine-2′-phenylindoldihydrochloride (DAPI; Roche Diagnostics GmbH, Mannheim, Germany) fluorescence counterstain in accordance with the manufacturer’s instructions. For this purpose, about 4000 cells/cm^2^ were cultivated for 48 hours in the corresponding media before fixation and SA-β-gal staining. After completion of the staining procedure, four representative images were taken from diverse areas of each cell culture using phase-contrast microscopy and Cell^B^ Imaging Software (Olympus GmbH, Hamburg, Germany). For the calculation of the percentage of senescent cells, the total number of cell nuclei and the number of cell nuclei surrounded by cyan dye were enumerated.

### Analysis of surface markers by flow cytometry

Characterization of the MSC immunophenotype was performed as described previously [7]. Continuously proliferating MSC were harvested and analyzed for cell surface marker expression by flow cytometry. After blocking nonspecific binding to Fc-receptors by incubation of 10^6^ cells with 2 % fetal calf serum (FCS) for 15 minutes at 4 °C and washing with PBS–bovine serum albumin (BSA), the cells were incubated with the following appropriately-labeled monoclonal anti-human antibodies: CD73-phycoerythrin (PE) (clone AD2; BD Bioscience, Heidelberg, Germany), CD90-PE (clone 5E10, IgG1; BioLegend Inc., San Diego, CA, USA), CD105-PE (clone 43A3, IgG1; BioLegend Inc.), CD14-fluorescein isothiocyanate (FITC) (clone TÜK4, IgG2a; MACS, Miltenyi, Bergisch-Gladbach, Germany), CD31-FITC (clone WM59, IgG1; DAKO, Hamburg, Germany), CD34-PE (clone AC136, IgG2a; DAKO), and CD45-FITC (clone T29/33, IgG1; DAKO). Following antibody staining, all samples were washed twice with PBS–BSA. Positive staining was obtained according to control measurements of the different populations with isotype-matching IgG control antibodies. Flow cytometry analysis and histograms were performed in a Galaxy FACSan (Partec) using FloMax analysis software (Partec).

### Cell differentiation

Osteogenesis was induced by maintaining confluent cell cultures in Dulbecco’s modified Eagle’s medium (DMEM: Dulbecco's Modified Eagles's Medium)/F12 supplemented with 0.15 mM ascorbat-2-phosphate, 0.1 μM dexamethasone, 3 mM β-glycerol, 10 % fetal bovine serum (FBS; Biochrom, Berlin, Germany), and 1 % penicillin–streptomycin for 4 weeks. Differentiation was visualized by light microscopy and Alizarin red staining.

For adipogenic differentiation the different MSC cultures were first grown to confluence and then cultured in the appropriate differentiation medium (DMEM/F12 with l-glutamine (PAA, Cölbe, Germany) with 10 % FBS, 1 % penicillin–streptomycin, 0.15 mM ascorbat-2-phosphate, 1 μM dexamethasone, 0.5 mM 3-isobutyl-1-methylxanthine, 0.1 mM indomethacin, and 4 U/l insulin (Aventis, Frankfurt, Germany)) for 14 days, respectively. The maturity of an adipocyte phenotype was evaluated by light microscopy and oil red O staining.

For chondrogenic differentiation, cells were kept in pellet culture after centrifugation at 300 × *g* for 5 minutes. The cultures were cultivated in DMEM/F12 supplemented with 0.15 mM ascorbat-2-phosphate, 1 % insulin, transferrin, selenium, ethanolamine solution (ITS-X; Life Technologies, Darmstadt, Germany), 100 mM sodium pyruvate (Biochrom), 0.1 μM dexamethasone, 0.35 mM proline, and 10 ng/ml TGFβ1 (Peprotec, Rocky Hill, NJ, USA) for 3 weeks. Afterwards, the pellets were rinsed twice in PBS and fixed in 4 % formaldehyde in PBS, embedded in paraffin, and cut into sections of 5 μm thickness. The sections were stained with alcian blue for detection of glycosaminoglycans.

## Results

**Fig. 1:**
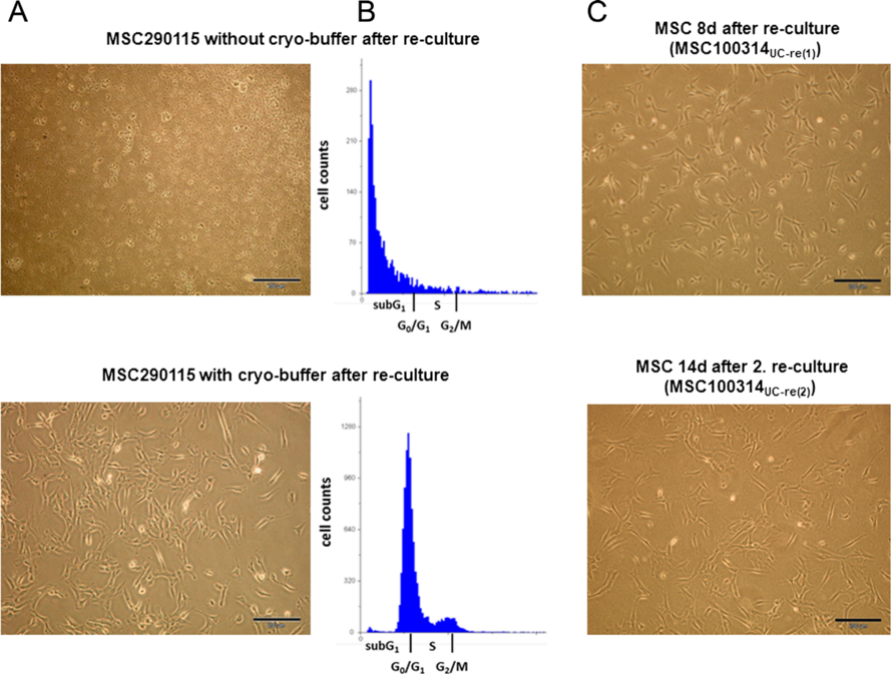
Morphology and cell cycle properties of recultured UC tissue. **a** Cryopreserved pure UC290115 tissue pieces in liquid nitrogen without cryobuffer or any other additives (*upper panel*) and in the presence of cryobuffer (*lower panel*) were recultured in MSC medium for 14 days and the morphology of potential MSC was documented (magnification bars = 100 μm). **b** Potential MSC from 14-day explant cultures of recultured and previously cryopreserved UC290115 tissue pieces in liquid nitrogen without cryobuffer (*upper panel*) and in the presence of cryobuffer (*lower panel*) were harvested and subjected to cell cycle analysis. Distribution within sub-G1, G0/G1, S, and G2/M cell cycle phases was performed by FloMax cell cycle software. **c** Reculture of conditioned UC100314_re(1)_ tissue pieces after cryopreservation in liquid nitrogen with cryobuffer revealed the outgrowth of MSC-like cells (MSC100314_UC-re(1)_) within 8 days (*upper panel*). In addition, a second reculture after repeated cryopreservation of these UC tissue pieces (UC100314 _re(2)_) demonstrated an explant culture of MSC-like cells (MSC100314 _UC-re(2)_) within 14 days (magnification bars = 100 μm). *MSC* mesenchymal stroma/stem cells

Direct cryopreservation of freshly prepared UC tissue pieces in liquid nitrogen without cryomedium and a following reculture in MSC medium was associated with the production of viscous material in the supernatant and appearance of debris and dead cells within 14 days (Fig. 1a, upper panel). Supportive evidence was obtained by cell cycle analysis of this culture demonstrating predominantly DNA fragments in the sub-G1 phase as an indication for cell death (Fig. 1b, upper panel). In contrast, reculture of UC tissue pieces previously cryopreserved in the presence of cryomedium revealed the initial production of viscous material and the outgrowth of MSC-like cells after 14 days (Fig. 1a, bottom panel), which was paralleled by a cell cycle of a proliferating population demonstrating cells in G0/G1, S, and G2/M phases (Fig. 1b, bottom panel).

Alternatively, direct explant culture of freshly prepared UC tissue for about 20 days was accompanied by initial outgrowth of MSC-like cells, whereby the UC tissue became “conditioned”. Liquid nitrogen cryopreservation of these conditioned UC tissue pieces with cryomedium followed by reculture exhibited an outgrowth of viable MSC-like cell populations already within 8 days (Fig. 1c, upper panel), whereby the first cells were observed within 2 days of reculture. These differences demonstrated that the outgrowth of cells from the conditioned UC tissues starts immediately after reculture, in contrast to freshly prepared UC tissue still requiring adaptation to the culture conditions. Moreover, a second cryopreservation of these recultured conditioned UC100314 tissue pieces in liquid nitrogen and another second reculture (UC100314-Re) was accompanied by a similar outgrowth of MSC-like cells within 14 days (Fig. 1c, bottom panel).

**Fig. 2:**
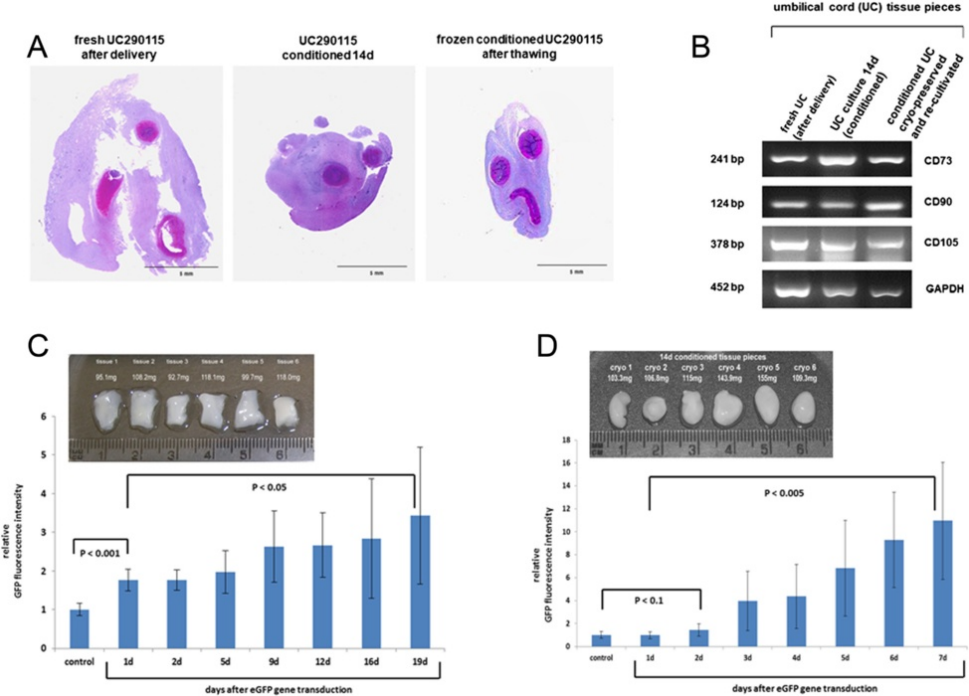
Histochemical analysis of fresh and conditioned UC. **a. ** Tissue section (4 μm) of hematoxylin and eosin-stained fresh UC tissue (*left panel*) was compared with conditioned UC tissue following a 14-day explant culture (*middle panel*) and with frozen conditioned UC tissue after thawing and reculture (*right panel*). **b** Expression of MSC markers in UC tissue pieces. mRNA from UC tissue pieces at different conditions (fresh UC, conditioned UC, and frozen conditioned UC after reculture) was analyzed by RT-PCR for transcripts of the MSC markers CD73, CD90, and CD105 with GAPDH expression as a control. **c** Proliferation of MSCs in fresh UC tissue pieces during conditioning. UC tissue pieces of similar size and weight (*graph insert*) prepared directly after delivery were labeled by lentiviral eGFP gene transduction. Thereafter, the UC tissues were incubated in MSC medium for up to 19 days and the GFP fluorescence intensity in the tissues was monitored at the time points indicated. Data represent the mean ± standard deviation (SD) (*n* = 6). Statistical analysis was performed by Student's *t* test between the fluorescence intensities of the unlabeled UC tissues (autofluorescence of the tissues = control) and after eGFP gene transduction (*p* <0.001). Moreover, statistical differences between the fluorescence intensities of day 1 and day 19 revealed a significance of *p* <0.05. **d** Proliferation of MSC in cryopreserved UC tissue pieces after reculture. UC tissue pieces of similar size and weight (*graph insert*) from conditioned UC tissue pieces after cryopreservation and reculture were labeled by lentiviral eGFP gene transduction. Thereafter, the UC tissues were incubated in MSC medium for up to 7 days and the GFP fluorescence intensity in the tissues was monitored every 24 hours. Data represent the mean ± SD (*n* = 6). Statistical analysis was performed by Student's *t* test between the fluorescence intensities of the unlabeled UC tissues (autofluorescence of the tissues = control) and after the second day of eGFP gene transduction (*p* <0.1). Moreover, statistical differences between the fluorescence intensities of day 1 and day 7 revealed a significance of *p* <0.005. *bp* base pairs, *GFP* green fluorescent protein

These findings underscored the use of cryomedium for tissue preservation and suggested that the process of UC tissue conditioning involved an intratissue remodeling to enable growth stimulation with protection of the new cells. Histochemical analysis after hematoxylin and eosin staining of UC tissue confirmed morphological changes by a marked condensation of the vessels and the connective tissue within the UC after 14 days of conditioning and in recultured UC tissue after cryopreservation as compared with fresh UC tissue (Fig. 2a).

The expression of MSC characteristic genes was also observed in the UC tissue pieces under the different conditions. RNA from fresh UC tissue as well as 14-day conditioned UC tissue or conditioned UC tissue after cryopreservation and reculture demonstrated transcripts for CD73, CD90, and CD105 (Fig. 2b).

The conditioning of UC tissue was investigated in six UC tissue pieces of approximately similar size and weight which were cultured for up to 19 days with prior GFP labeling of tissue-associated cells via lentiviral transduction. During this explant culture, progressively increasing GFP fluorescence intensities of the tissue pieces by 3.4 ± 1.7-fold (*n* = 6; *p* <0.05) were measured (Fig. 2c). Previous work has demonstrated that the GFP fluorescence intensities corresponded to an appropriate cell number [24]. Consequently, conditioning of UC tissue was associated with a significant increase of proliferating cells within the tissue. To further address this hypothesis, six conditioned UC tissue pieces after cryopreservation were recultured and similarly GFP labeled. The conditioned tissue pieces were more round-shaped (Fig. 2d) compared with the initial freshly prepared tissue as observed previously [5]. Already within 7 days of explant culture the fluorescence intensity of the conditioned tissue pieces increased by 11.0 ± 5.1-fold (*n* = 6: *p* <0.005), which substantiated enhanced and accelerated cell growth after tissue conditioning (Fig. 2d) as compared with the initial fresh culture that took about 19 days with a much lower fluorescence intensity (Fig. 2c).

**Fig. 3:**
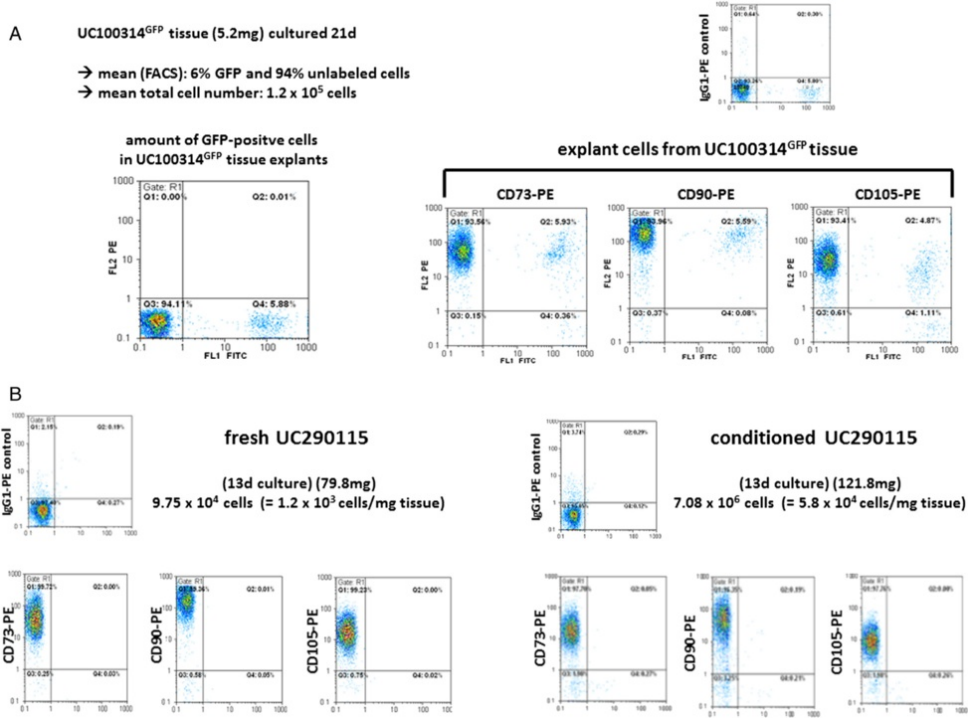
Cell yield and surface marker analysis of explants from GFP-labeled UC tissues. **a** Quantification of total cell number and GFP-positive cells after explant culture of GFP-labeled UC tissue was performed by flow cytometry (*upper panel*). The outgrowing cells after 21 days were analyzed for the PE-labeled MSC-specific markers CD73, CD90, and CD105, demonstrating the amount of PE-positive unlabeled cells and the appropriate amount of antibody-positive and GFP-positive cells within the double fluorescence channel, respectively (*lower panel*). **b** Comparison of the amount of outgrowing cells with the MSC-like characteristic surface marker expression CD73, CD90, and CD105 from fresh (*upper panel*) and conditioned (*lower panel*) UC tissue was quantified after 13 days of explant culture and calculated as cell yield per milligram of tissue. *FACS* fluorescence-activated cell sorting, *FITC* fluorescein isothiocyanate, *GFP* green fluorescent protein, *PE* phycoerythrin

Following 21 days of explant culture, quantification and phenotyping of the outgrowing cells from 5.2 mg GFP-transduced UC tissue were performed by flow cytometry analysis. A total yield of 1.2 × 10^5^ cells contained about 6 % of GFP-positive cells (Fig. 3a, left panel). Flow cytometry evaluation of the MSC-typical markers CD73, CD90, and CD105 always revealed in total more than 98 % positive cells, whereby about 93–94 % exhibited a PE signal of the unlabeled cells. The remaining about 5–6 % reflected the outgrowing cells with a double fluorescence of PE and GFP (detected by the FITC channel) in accordance with the transduced population (Fig. 3a, right panel).

A direct comparison between fresh and conditioned UC tissue substantiated an advantage of the conditioning whereby all outgrowing cells from both tissues expressed the MSC-typical markers CD73, CD90, and CD105 (Fig. 3b). A 13-day explant culture revealed about 1.2 × 10^3^ cells/mg fresh UC tissue (Fig. 3b, left panel) whereas prior conditioning yielded about 5.8 × 10^4^ cells/mg conditioned tissue (Fig. 3b, right panel).

Together, these findings demonstrated that cells including GFP-labeled populations are proliferating within the UC tissue during conditioning. Consequently, conditioned UC tissue represented an accelerated initial outgrowth during explant culture whereby explanted cells exhibit MSC-like properties.

**Fig. 4:**
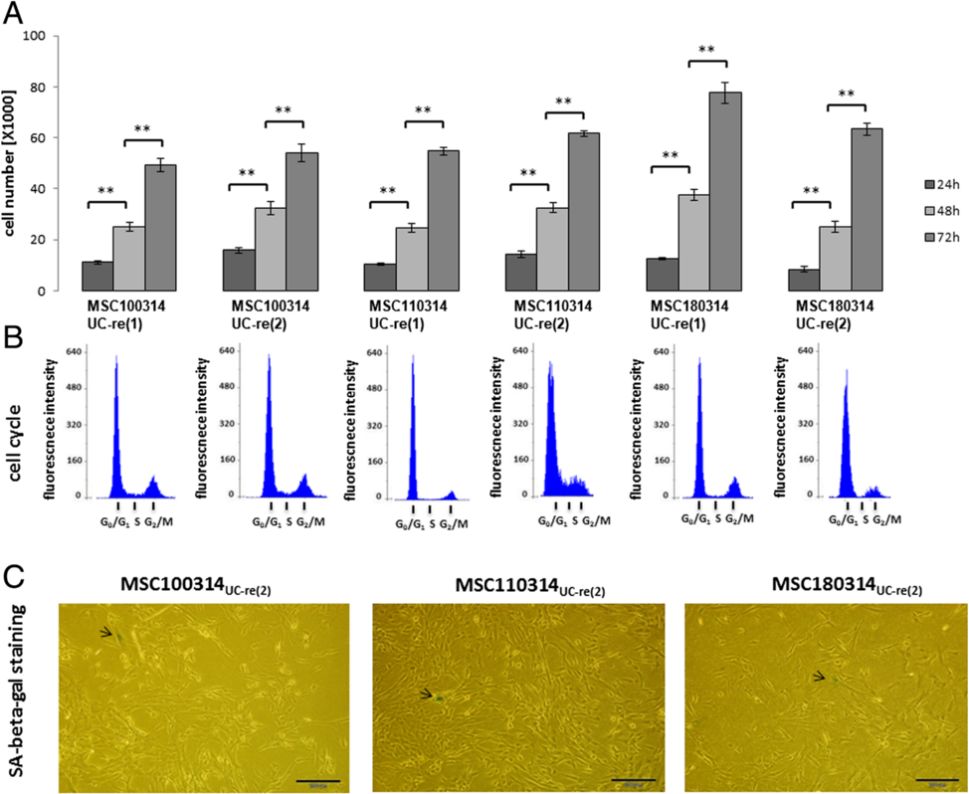
Proliferative capacity of conditioned UC tissue-derived cells. **a** Proliferation of MSC-like cells obtained after reculture of cryopreserved conditioned UC tissues from three different donors (MSC100314_UC-re(1)_, MSC110314_UC-re(1)_, MSC180314_UC-re(1)_) was evaluated by cell counting in a hemocytometer after initial seeding of 10^4^ cells from each MSC population. Relative proliferative capacity was calculated as fold changes after setting the initial cell number 10^4^ cells = 1. Likewise, a second reculture after repeated cryopreservation of these UC tissue pieces (MSC100314_UC-re(2)_, MSC110314_UC-re(2)_, MSC180314_UC-re(2)_) demonstrated similar cell growth properties compared with the previous explant culture of the same tissues under the same experimental conditions. Data represent the mean ± SD of three independent experiments. Statistical analysis was performed by Student’s *t* test (**p <0.001). **b** A parallel cell cycle analysis of the MSC explant cultures was performed by flow cytometry and demonstrated proliferating cells in all cultures by the appearance of all cell cycle phases in the histograms. **c** Detection of senescent cells in MSC_UC-re(2)_ explant culture by SA-β-gal staining. Staining of outgrowing MSC-like cells (MSC100314_UC-re(2)_, MSC110314_UC-re(2)_, MSC180314_UC-re(2)_) obtained from the repeated reculture of a second corresponding tissue cryopreservation with the SA-β-gal kit demonstrated only a marginal expression of this aging marker (*arrows*) in less than 2 % of the cells. *SA-β-gal* senescence-associated β-galactosidase

Cells obtained after reculture of conditioned and cryopreserved UC tissues from three individual donors were further analyzed for stem cell characteristics including proliferative capacity, expression of MSC markers, and differentiation potential along mesenchymal lineages. The three primary cultures MSC100314_UC-re(1)_, MSC110314_UC-re(1)_, and MSC180314_UC-re(1)_, obtained from corresponding cryopreserved and recultured UC tissues, respectively, exhibited significant proliferative activity (Fig. 4a). Moreover, cells from the second reculture after repeated cryopreservation of the same tissues (MSC100314_UC-re(2)_, MSC110314_UC-re(2)_, MSC180314_UC-re(2)_) demonstrated similar cell growth potential compared with the explant cultures from the first UC reculture (Fig. 4a). Likewise, all six populations revealed continuous cell cycle progression and little if any differences were detectable in the pattern and distribution of cell cycle phases between cell populations from the first or the second reculture of UC tissues (Fig. 4b). These data pointed to a constant growth capacity of cells irrespective of one or two freezing cycles of the originating conditioned tissue. Further support was obtained by the measurement of SA-β-gal, with less than 2 % of positive cells in all cultures indicating that even after the second reculture of cryopreserved conditioned UC tissue a majority of outgrowing juvenile and proliferative active cells prevailed (Fig. 4c).

**Fig. 5:**
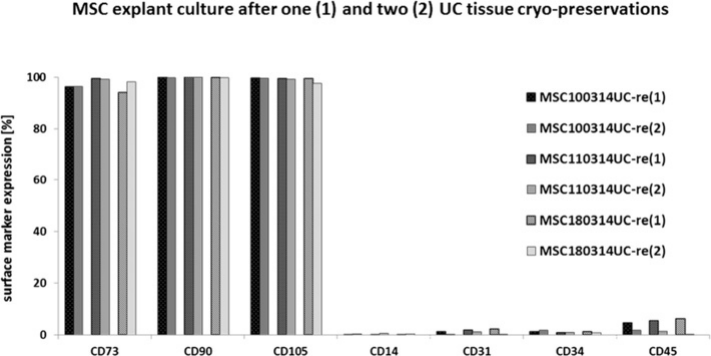
MSC surface marker analysis. Cell surface marker evaluation was performed for the presence of the typical MSC markers (CD73, CD90, and CD105) by flow cytometry analysis in cells of three individual UC explant cultures of the previously cryopreserved UC tissues. **a** MSC100314_UC-re(1)_. **b** MSC110314_UC-re(1)_. **c** MSC180314_UC-re(1)_. Moreover, the MSC marker expression was compared with cells obtained after second reculture following repeated cryopreservation of these UC tissue pieces. **d** MSC100314_UC-re(2)_. **e** MSC110314_UC-re(2)_. **f** MSC180314_UC-re(2)_. A simultaneous absence of further cell type-specific surface markers CD31, CD34, CD45, and CD14 was tested. The analysis was performed in steady state-growing cells by flow cytometry compared with the corresponding immunoglobulin isotype antibodies which were used as appropriate controls in the histograms. *MSC* mesenchymal stroma/stem cells

MSC characteristics in these explant populations were evaluated by the presence of CD73, CD90, and CD105, respectively. Flow cytometry analysis demonstrated always more than 90 % of these MSC-associated surface markers in MSC100314_UC-re(1)_, MSC110314_UC-re(1)_, and MSC180314_UC-re(1)_, as well as in corresponding cell populations derived from reculture of the second cryopreservation (MSC100314_UC-re(2)_, MSC110314_UC-re(2)_, MSC180314_UC-re(2)_) (Fig. 5), respectively. These findings were paralleled by little if any detectable CD14, CD31, CD34, or CD45 surface markers in either population (Fig. 5), further substantiating the outgrowth of MSC-like cells in the explant cultures.

**Fig. 6:**
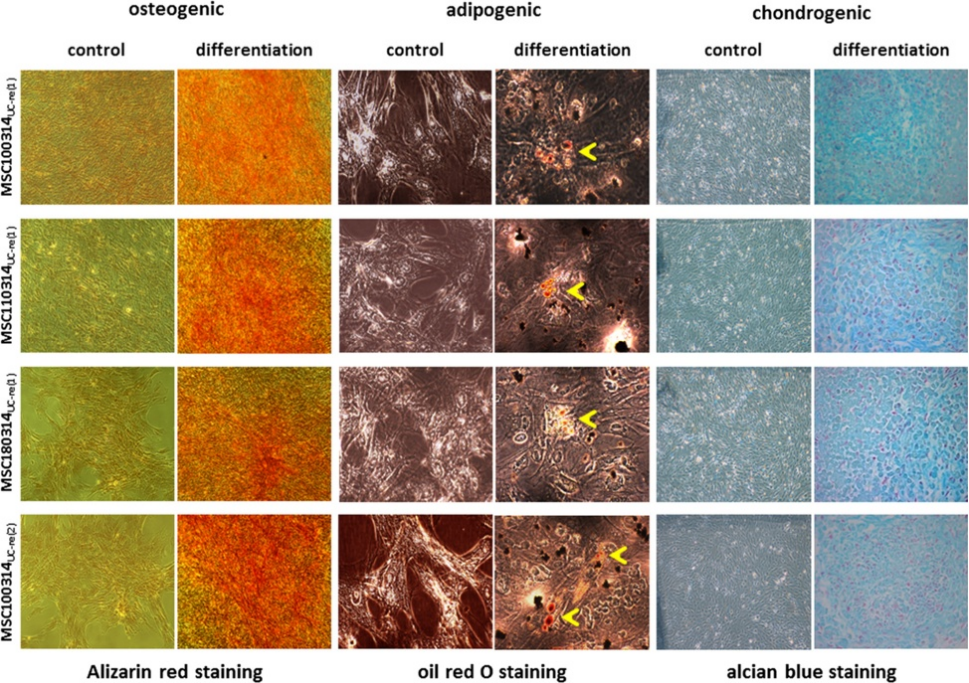
Differentiation capacity of conditioned UC tissue explant cultures. After thawing and reculture of three conditioned cryopreserved UC tissues as well as second thawing and repeated reculture (MSC100314_UC-re(2)_) of previously cryopreserved UC tissue, the resulting MSC explant cultures were investigated for their potential to differentiate along the osteogenic, adipogenic, and chondrogenic pathway. All explant cultures when maintained in appropriate differentiation medium demonstrated strong Alizarin red staining which indicated increased calcification during osteogenesis in contrast to MSC control cultures maintained in normal growth medium. Moreover, the appearance of lipid droplets in the cultures as indicated by oil red O staining (*yellow arrowheads*) revealed adipogenic properties. Finally, enhanced production of proteoglycans within the extracellular matrix represents a property of chondrogenic cells which was detectable by alcian blue staining of differentiated MSC in contrast to corresponding control cells

Stem cell properties of MSC also include the capability to differentiate at least along the osteogenic, adipogenic, and chondrogenic lineage. Induction of this tri-lineage maturation revealed strong similarities in the differentiation potential of the MSC100314_UC-re(1)_, MSC110314_UC-re(1)_, and MSC180314_UC-re(1)_ explant cultures and from the second reculture after repeated cryopreservation (MSC100314_UC-re(2)_, (Fig. 6). Thus, mineralization of the explant cultures assayed by Alizarin red staining demonstrated osteogenic potency in contrast to undifferentiated controls (Fig. 6, left panels). Moreover, adipogenic potential is usually demonstrated by increased lipid metabolism associated with the formation of intracellular lipid droplets stained with oil red O which could be observed exclusively in the adipogenic differentiation-induced explant populations (Fig. 6, middle panels). A variety of sulfated proteoglycan deposits are properties of functional chondrocytes which can be determined by alcian blue, and significantly enhanced staining was detected in the differentiated cultures compared with the undifferentiated controls (Fig. 6, right panels).

**Fig. 7:**
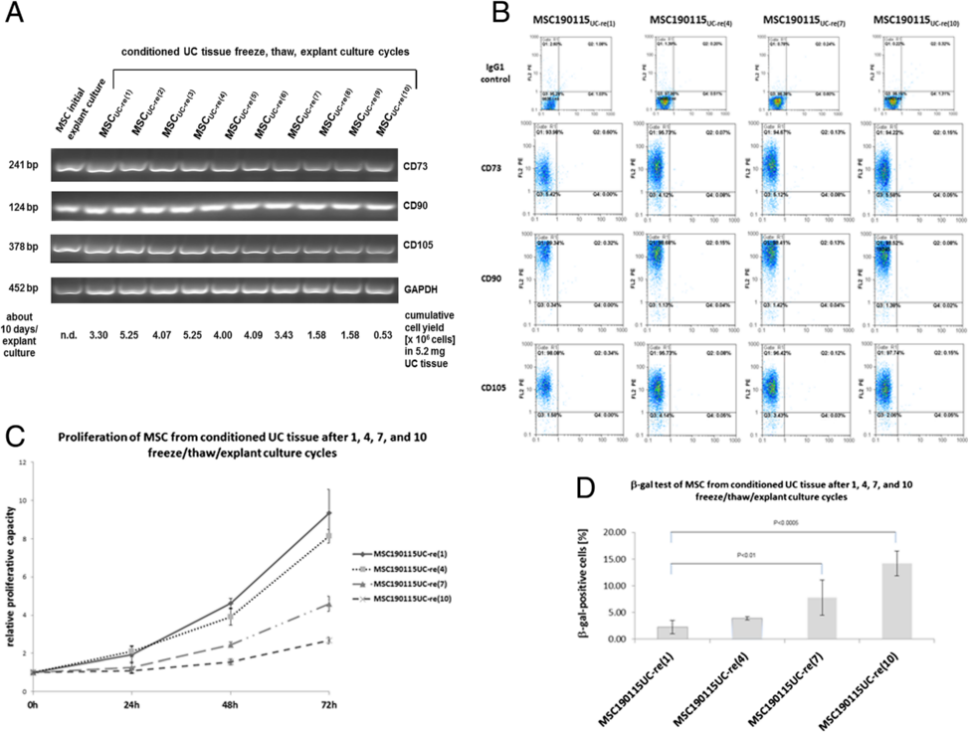
Characterization of MSCs after repeated freeze/thaw/explant cultures. **a** MSC marker expression in repeated freeze/thaw/explant cultures. Conditioned UC tissue after initial explant culture was subjected to 10 cycles of freeze/thaw/explant culture and cell cultures obtained from each cycle were harvested and analyzed by RT-PCR for the expression of the MSC markers CD73, CD90, and CD105 with GAPDH expression as a control. The cumulative yield of MSC from each freeze/thaw/explant culture cycle was calculated for a 10-day period. **b** Conditioned UC tissue after initial explant culture was subjected to one, four, seven, and 10 freeze/thaw/explant culture cycles, respectively, and cell surface marker analysis was performed for the presence of the typical MSC markers (CD73, CD90, and CD105) by flow cytometry. **c** Proliferative capacity of MSC-like cells obtained after reculture of cryopreserved conditioned UC tissues from one, four, seven, and 10 freeze/thaw/explant culture cycles was evaluated by cell counting in a hemocytometer after initial seeding of 10^4^ cells from each MSC population. Relative proliferative capacity was calculated as fold changes after setting the initial cell number 10^4^ cells = 1. Data represent the mean ± SD of four independent experiments. Statistical analysis was performed by Student’s *t* test. **d** Conditioned UC tissue after initial explant culture was subjected to one, four, seven, and 10 freeze/thaw/explant culture cycles, respectively, and the SA-β-gal assay was applied to the outgrowing MSC cultures. SA-β-gal-positive cells were quantified as mean ± SD of three independent experiments. Statistical analysis was performed by Student’s *t* test. *bp* base pairs, *MSC* mesenchymal stroma/stem cells, *PE* phycoerythrin, *UC* umbilical cord

Maintenance of MSC stemness by repeated freeze/thaw/explant culture cycles of UC tissue revealed an unaltered cell morphology (data not shown). Moreover, all cultures demonstrated consecutive expression of the MSC-specific markers CD73, CD90, and CD105 starting from the first explant culture during conditioning of MSC tissue until the 10th cryopreservation of the same UC tissue with subsequent thawing and explant reculture (MSC_UC-re(10)_) (Fig. 7a). These findings were also substantiated by flow cytometry analysis demonstrating expression of CD73, CD90, and CD105 in more than 90 % of selective populations every third freeze/thaw/explant culture cycle, respectively (Fig. 7b). The proliferative capacity of these MSC from conditioned UC tissues after one, four, seven, and 10 freeze/thaw/explant culture cycles revealed little if any differences between one and four freeze/thaw/explant culture cycles; however, seven and 10 freeze/thaw/explant culture cycles were associated with a significantly reduced proliferative capacity (Fig. 7c). These findings were substantiated by determination of the MSC yield from consecutive 10-day UC tissue explant cultures demonstrating a progressive decrease of outgrowing MSC after six freeze/thaw/explant culture cycles (Fig. 7a). The data demonstrated an accumulated yield of about 3.3 × 10^7^ MSC from 5.2 mg UC tissue within 10 days. Based on an average UC length of 50–60 cm with 1.5–2 cm diameter and 41 g weight, an optimal primary explant culture using the whole UC tissue could initially yield about 2.6 × 10^11^ MSC. In the case of fresh UC tissue, conditioning during explant culture would last about 2–3 weeks, and thereafter continuous explant culture of these tissue pieces from the whole UC could yield a total amount of more than 10^10^ MSC within 4 weeks. Moreover, these cells can be subcultured from each explant culture in various distinct passages to further potentiate the number of MSC.

During consecutive freeze/thaw/explant culture cycles, potential stem cell aging was quantified by the SA-β-gal assay. Whereas low levels of SA-β-gal-positive cells were observed in MSC_UC-re(1)_ as 2.8 ± 1.3 % and in MSC_UC-re(4)_ cultures as 3.9 ± 0.3 %, these numbers progressively and significantly increased in populations of MSC_UC-re(7)_ to 7.8 ± 3.3 % and in MSC_UC-re(10)_ to 15.3 ± 0.5 % (Fig. 7d). Together, these findings suggested that the reduced proliferative capacity of MSC derived from 10 freeze/thaw/explant culture cycles correlated with significantly elevated amount of SA-β-gal-positive cells.

**Fig. 8:**
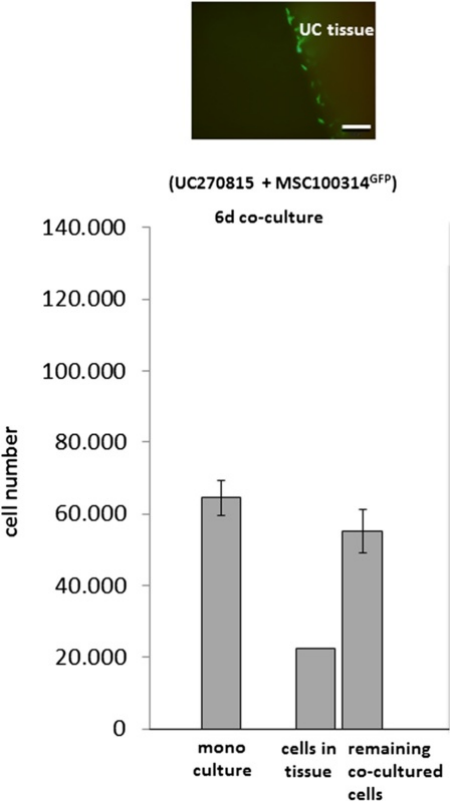
Analysis of MSC homing to UC tissues. Coculture of about 100 mg conditioned UC tissue without further MSC outgrowth (*n* = 4) was performed together with GFP-labeled MSCs seeded at 1000 cells/cm^2^ in comparison with a monoculture of MSC^GFP^ at the same cell density. After 6 days of coculture, the UC tissue was removed, washed three times in PBS, and documented by fluorescence microscopy. Moreover, the UC tissue and the remaining originally cocultured cells as well as the corresponding monoculture of MSC^GFP^ were lysed by the addition of SDS and appropriate fluorescence intensities were quantified in aliquots using a Fluoroskan Ascent FL microtiter plate reader and calculated as mean ± SD (*n* = 4) for the amount of cells according to a standard curve of GFP-labeled MSC. *UC* umbilical cord

In order to address a potentially preferred homing of MSC to UC tissue rather than plastic, UC270815 tissue (96.3 ± 50.1 mg/tissue; *n* = 4) was cocultured with MSC100314^GFP^ (1000 cells/cm^2^) in four separate experiments, whereby the original outgrowth of cells from these tissues had stopped. This coculture was performed on plastic in 24-well plates (Nunc) and cell growth was compared with a corresponding MSC monoculture at the same experimental conditions. After 6 days of culture, the UC tissue and the additional cells in the well and also the monoculture cells were lysed by SDS and aliquots were measured for fluorescence intensities and calculated to the appropriate amount of cells. Whereas 6.65 ± 0.48 × 10^4^ MSC^GFP^ were detected in the monoculture, the coculture was associated with 5.51 ± 0.62 × 10^4^ MSC^GFP^ attached to the plastic in the well and an additional 2.26 × 10^4^ MSC^GFP^ were associated with the UC tissue (Fig. 8). These findings suggested enhanced cell growth in the presence of UC tissue and a preferred localization of MSC^GFP^ in the UC tissue since the amount of MSC plastic adherence in the UC tissue coculture was markedly lower as compared with the MSC plastic adherence in the monoculture. Indeed, a fluorescence micrograph confirmed incorporation of MSC^GFP^ into the rim of the UC tissue (Fig. 8). Additional preliminary observations were obtained following three extensive washes with PBS and further culture of the MSC^GFP^-incorporated UC tissue (61.6 mg) in a separate new well for 7 days, which was associated with a significantly enhanced proliferation reaching 1.2 × 10^5^ MSC^GFP^ within the tissue similar to a conditioning of the tissue. Moreover, about 10^3^ cells were detected as explant culture from this tissue. These preliminary findings were substantiated by a fluorescence micrograph demonstrating the whole UC tissue filled with MSC^GFP^ and additional outgrowth of some cells (data not shown) which indicated conditioning of the UC tissue.

## Discussion

Multipotent human MSC represent a preferred tool for cell or tissue replacement therapy and are involved in a large variety of clinical studies in regenerative medicine. To meet the progressively increasing requirement of MSC as a more frequently used stem cell population in tissue engineering, UC provides a noninvasive, easily accessible, and ethically noncontroversial MSC-rich stem cell source [4]. Moreover, cryostorage of MSC-originating tissue significantly elevates the availability of reproducible material and potential cryoconditions for tissues and other biospecimens have been tested previously [28]. Accordingly, cryopreservation of conditioned UC tissue in liquid nitrogen demonstrated a constant availability of MSC with reproducible properties including proliferative capacity, the maintenance of certain plasticity, and stable stem cell characteristics.

All MSC obtained after reculture of previously cryopreserved UC tissues from various donors demonstrated at least differentiation capacity along mesodermal lineages, including acquisition of osteogenic, adipogenic, and chondrogenic properties. Moreover, UC tissue and the corresponding explant cultures expressed CD73, CD90, and CD105, representing the minimal criteria for MSC markers. Previous work demonstrated that some MSC subpopulations also carry STRO-1 [29], or the receptors vascular cell adhesion molecule-1 (VCAM-1, CD106) and intercellular cell adhesion molecule-1 (ICAM-1, CD54) [30] preferably expressed in bone marrow-derived MSC. In addition, octamer-binding transcription factor-4 (OCT-4) and (sex determining region Y)-box-2 (SOX2) as embryonic-like stem cell markers can be detected in certain MSC subpopulations [31], further substantiating MSC heterogeneity and plasticity by the appearance, acquisition, or loss of distinct properties. Furthermore, MSC contribute to a transfer and exchange of markers with neighboring tissue cells, thereby altering cellular functionality by enhanced plasticity [25, 32, 33].

Conditioning of UC tissue prior to cryopreservation by initial culture for about 2–3 weeks demonstrated proliferation of cells within the tissue to reach certain intratissue saturation. This was followed by active outgrowth of cells carrying typical MSC markers during explant culture.

Different isolation protocols to obtain MSC from UC tissue include enzymatic digestion of the extracellular matrix by either trypsin, collagenase, or accutase treatment, in contrast to another technique which is keeping an intact and physiological extracellular matrix by explant culture of MSC from UC tissue pieces. Whereas certain results favor enzymatic tissue treatment [34], other studies demonstrated an advantage of MSC explant culture [35]. However, differences in MSC culture conditions including various media and, more importantly, the use of poorly defined additives such as FCS instead of xeno-free components further contribute to heterogeneity of the data.

Previous work has suggested that the enzyme-free explant culture ensures the maintenance of an intact microenvironment within the tissue pieces to keep a natural three-dimensional stroma structure for MSC with appropriate surface interaction sites [5]. Interestingly, this MSC-like outgrowth during explant culture was significantly accelerated after reculture of cryopreserved conditioned UC tissue when the UC tissue structure became more condensed by contracting forces. Other studies observed a gradient of cell maturity within the UC tissues [36] and considered the cytoskeletal complexity in UC tissue, which supports the idea of a location-dependent and microenvironment-dependent growth and differentiation potential of UC-derived MSC [36, 37].

In addition to the results of the UC tissues in this study, storage of MSC cultures can also be performed by cryopreservation in liquid nitrogen [38]. However, subculture of MSCs is associated with limited expansion until approximately 10 passages [5, 39]. Similar findings were obtained during repeated freeze/thaw/explant culture cycles whereby reduced proliferative capacity after 10 cycles was accompanied by elevated senescence in the culture as determined by an increased amount of SA-β-gal-positive cells. In addition, previous work has demonstrated prolonged culture of MSC by keeping the originating UC tissue structure to provide a more physiological three-dimensional microenvironment for explant culture [5]. Indeed, the comparison of MSC culture on plastic versus UC tissue demonstrated a preferred homing of MSC to UC tissue.

## Conclusion

In summary, these findings suggested that maintenance of MSC in a three-dimensional culture enables the establishment of a natural microenvironment providing long-term outgrowth potential of stem cell-like cells with unchanged growth properties for at least four freeze/thaw/explant culture cycles. Moreover, repeated cryopreservation of conditioned UC tissues keeps stroma/stem cell multiplicity and thereby minimizes a likely culture condition-mediated clonal selection or progressive clonal convergence towards an accumulation of certain subpopulations. Consequently, these tissues provide a microenvironment for maximal plasticity with reasonable amounts of originating MSC which is required in the field of regenerative medicine. Moreover, this more natural three-dimensional stem cell culture also enables UC tissue banking with reproducible availability of MSC for cell therapies and tissue engineering.

## Abbreviations

αMEM: Minimal Essential Medium Eagle, alpha-modification
bp: Base pairs
BSA: Bovine serum albumin
DAPI: 4′,6-Diamidine-2′-phenylindoldihydrochloride
DMEM: Dulbecco’s modified Eagle’s medium
DMSO: Dimethyl sulfoxide
FBS: Fetal bovine serum
FCS: Fetal calf serum
FITC: Fluorescein isothiocyanate
GAPDH: Glyceraldehyde 3-phosphate dehydrogenase
GFP: Green fluorescent protein
HLA-DR: Human leukocyte antigen DR
HS: Human AB-serum
ICAM-1: Intercellular cell adhesion molecule-1
MSC: Mesenchymal stroma/stem cells
OCT-4: Octamer-binding transcription factor-4
PBS: Phosphate-buffered saline
PE: Phycoerythrin
RT: Reverse transcription
SA-β-gal: Senescence-associated β-galactosidase
SD: standard deviation
SOX-2: (Sex determining region Y)-box-2
UC: Umbilical cord
VCAM-1: Vascular cell adhesion molecule-1
